# *mSphere* of Influence: There’s More to (a Pathogen’s) Life than Growing Fast

**DOI:** 10.1128/msphere.00277-22

**Published:** 2022-07-07

**Authors:** Nina Wale

**Affiliations:** a Department of Microbiology & Molecular Genetics, Michigan State University, East Lansing, Michigan, USA; b Department of Integrative Biology, Michigan State University, East Lansing, Michigan, USA; c Program in Ecology, Evolution and Behavior, Michigan State University, East Lansing, Michigan, USA

**Keywords:** evolution, microbial ecology, parasite, pathogen, resource, trait, virulence

## Abstract

Nina Wale works in the field of infectious disease evolution and ecology. In this mSphere of Influence article, she reflects on how the paper by Roller and Schmidt, “The physiology and ecological implications of efficient growth” (B. R. Roller and T. M. Schmidt, ISME J 9:1481–1487, 2015, https://doi.org/10.1038/ismej.2014.235) broadened her thinking about how microbes acquire and allocate resources and, in so doing, set her research on pathogen virulence evolution in a new direction.

## COMMENTARY

The strategies that pathogenic microbes (pathogens, hereafter) use to acquire resources are a critical determinant of pathogen and host fitness. Pathogens are parasites. They steal resources from their hosts and use them to proliferate and, ultimately, transmit. By thus exploiting their hosts, pathogens reduce host fitness, i.e., they are virulent. Virulence can be costly to pathogens when it comes at the cost of transmission, e.g., when pathogens kill or immobilize their hosts. To maximize their fitness, therefore, pathogens must tune their exploitation of hosts to minimize the costs of virulence and maximize transmission. Classical models of virulence evolution (as well as popular opinion) assume that the relationship between virulence and transmission is mediated by a single pathogen trait: replication, or growth rate (1–3). Accordingly, fast-growing pathogens are better able to rapidly reach a transmissible density, but they are also more virulent than slower-growing ones. You can therefore imagine my surprise and confusion on reading the article by Roller and Schmidt, “The physiology and ecological implications of efficient growth” (4), which introduced to me another trait that connects microbial resource exploitation and fitness: growth efficiency. Here, I describe this paper’s contents and its influence on my thinking about pathogen resource exploitation and evolution.

Growth efficiency is defined as the number of progeny an organism produces for every resource it consumes. In their paper, Roller and Schmidt explored efficiency as a determinant of microbial fitness, i.e., as a life history trait. First, they reviewed experimental evidence for a trade-off between efficiency and (maximum) growth rate and identified ecological conditions that select for efficient microbes over fast-growing ones, e.g., low resource availability and flux, spatial heterogeneity. Second, they used a simple model and *in vitro* data to show that the efficiency of individual microbes is an increasing, saturating function of resource quantity and quality. This relationship arises because as resources increase in quantity and quality it is easier for microbes to meet the costs of maintenance, and so the proportion of resources that can be allocated to new biomass increases, i.e., growth efficiency increases. Finally, Roller and Schmidt made predictions about how the growth efficiency of two, distinct groups of free-living microbes, oligotrophs and copiotrophs, vary with resource availability. They began by noting the striking similarity between oligotrophic environments and the conditions that select for efficiency. They proposed that oligotrophs (i) begin growing, (ii) achieve maximally efficient growth, and (iii) cease to grow, at lower resource concentrations than copiotrophs. In addition, they speculated that oligotrophs can reach higher population sizes than copiotrophs. Critically, Roller and Schmidt provide a mechanistic rationale for their hypotheses: oligotrophs have reduced their investment in functions associated with maintenance, e.g., stress responses, motility, genome length. Thus, oligotrophs have lower “maintenance costs” and can grow in conditions that copiotrophs cannot; oligotrophs lose, however, in resource-rich environments where copiotrophs are able to meet their high maintenance costs.

This paper helped me make sense of evidence that, contrary to theoretical predictions, virulent pathogens do not grow fast (or, to be more precise, it put me on the path to making sense of this evidence). The slow growth rate of Mycobacterium tuberculosis is a constant source of frustration among my colleagues who study tuberculosis, which kills more people annually than any other infectious disease barring COVID-19. Their gripes seeded my doubts in the orthodox notion that pathogen growth rate is positively correlated with virulence. These doubts were reinforced by an analysis of 61 bacterial pathogens, which showed that growth rate was in fact negatively related to virulence (5). If growth rate does not mediate the relationship between pathogen resource exploitation, virulence, and transmission, what does? Roller and Schmidt’s paper offers one alternative. If virulence has a negative relationship with growth rate, then it should have a positive relationship with efficiency (assuming the rate versus efficiency trade-off holds among pathogens). One can imagine how such a relationship might emerge. If virulence is determined by a pathogen’s maximal population size, rather than the speed at which they reach it (as is often assumed [6]), efficient pathogens will be more virulent than fast-growing ones. Furthermore, efficient pathogens might have a competitive advantage over fast growers in some within-host environments. One of the first things the immune system does upon infection is reduce resource availability in tissues, a phenomenon called “nutritional immunity” (7). Depending on the degree of resource limitation, we might expect efficient pathogens to outcompete fast-growing ones in resource-depleted tissues. Unfortunately, however neat this solution might be, the idea that efficient pathogens are more virulent than fast-growing pathogens runs counter to classical theory. According to the assumptions of classical models, efficient pathogens will get a bigger bang (transmission) for their buck (virulence) and will thus evolve to a lower level of virulence.

My lab is working to resolve this quandary and, more generally, to better understand virulence evolution by drawing on key lessons provided by Roller and Schmidt. The first lesson is that the dynamics of resources in microbial habitats are a critical driver of microbial life history strategies. In the light of this lesson, the fact that resource dynamics within hosts are usually abstracted out of models of virulence evolution looks like an omission. We are thus working with theoretical ecologists to develop a model of virulence evolution that explicitly describes the dynamics of resources within hosts and their exploitation and investment by pathogens. This brings me to a second, more specific lesson of the Roller and Schmidt 2015 paper: to better understand pathogen evolution, we must characterize both the availability and the flux of resources within hosts. “Resource limitation” is an oft-invoked concept in disease ecology, and nutritional immunity is widely accepted in infectious disease biology, more broadly. In contrast to these ideas, which pertain to the availability of resources, resource flux has received much less attention. Recent studies suggest that the immune response to intracellular pathogens involves increasing the flux of cells that intracellular pathogens use as resources (8, 9), and tissue turnover rate is thought to mediate tolerance to infection (10). Roller and Schmidt’s paper suggests that such immune-mediated fluxes could play a role in shaping the different life histories of intracellular pathogens that target different tissues. Tantalizingly, new data indicate that the cellular population targeted by fast-growing malaria parasites (red blood cells) is much larger and has a higher rate of flux than that targeted by slow-growing M. tuberculosis (alveolar macrophages). Critically, to properly examine the role that resource flux has in shaping pathogen traits, we need to quantify resource dynamics in infected tissues during an infection. My lab is working to develop methods to achieve this goal. Together with our theoretical work, we thus aim to better understand the relationship between pathogens’ parasitic lifestyle and their pathogenicity.
